# A Matched Influenza Vaccine Strain Was Effective in Reducing the Risk of Acute Myocardial Infarction in Elderly Persons: A Population-Based Study

**DOI:** 10.1097/MD.0000000000002869

**Published:** 2016-03-11

**Authors:** Shu-Yun Hsu, Fong-Lin Chen, Yung-Po Liaw, Jing-Yang Huang, Oswald Ndi Nfor, Day-Yu Chao

**Affiliations:** From the Graduate Institute of Microbiology and Public Health, College of Veterinary Medicine, National Chung-Hsing University (S-YH, D-YC); Division of Pediatric Cardiology, Department of Pediatrics, Chung Shan Medical University Hospital (F-LC); and Department of Public Health and Institute of Public Health, Chung Shan Medical University (Y-PL, J-YH, ONN), Taichung, Taiwan.

## Abstract

The aim of this study was to explore whether matched or mismatched strains of influenza vaccines (IVs) are beneficial at reducing the risk of acute myocardial infarction (AMI) in elderly persons.

Data were obtained from the Longitudinal Health Database 2005 (LHID 2005) which is maintained by the National Health Insurance Research Institute in Taiwan. The analytical data included individuals who were vaccinated with mismatched vaccines during the October 2007 to December 2007 season and individuals vaccinated with matched strains during the October 2008 to December 2008 season. All participants were 65 years of age and older. In this analysis, individuals were considered to be exposed if their records showed that they were vaccinated against influenza, and they were considered to be nonexposed if they were not vaccinated during these seasons. A Cox hazard model was used to estimate AMI hazard ratio.

This study enrolled 93,051 exposed and 109,007 unexposed individuals. The AMI hazards ratios (HRs) for the men and women exposed to mismatched vaccine (in 2007) were 0.990 (95% confidence interval [CI], 0.745–1.316) and 1.102 (95% CI: 0.803–1.513), respectively. Men exposed to matched vaccines (in 2008) had significant HRs (HR: 0.681; 95% CI: 0.509–0.912) while the HRs in the women were barely significant (HR: 0.737; 95% CI: 0.527–1.029).

AMI risk could be particularly reduced in men if the IV matches well with the circulating strains in elderly people 65 years of age and older.

## INTRODUCTION

Vaccination against influenza has been negatively associated with the development of new myocardial infarction (MI) in patients with chronic coronary heart disease.^1^ Some studies have drawn different conclusions in terms of the association between influenza vaccination and cardiovascular diseases.^2–5^ One study has shown that influenza vaccination could be associated with a 19% reduction in the rate of acute myocardial infarction (AMI).^6^ Different countries have used vaccine strains that differ from those recommended by the World Health Organization. There has been antigenic mismatch between the vaccine and wild-type circulating strain. The effectiveness of annual influenza vaccines (IVs) varies depending on the match of the vaccine to circulating strains.^7^ However, no study has focused on antigenic mismatch between the vaccine and wild-type circulating strains.^8^ To clarify whether IVs helped to reduce the risk of acute myocardial infection, we analyzed elderly persons exposed to mismatched strains in 2007, as well as those exposed to matched strains in 2008.

## MATERIALS AND METHODS

The overall patient data were obtained from the Longitudinal Health Database 2005 (LHID 2005), which is maintained by the National Health Insurance Research Institute in Taiwan. The medical claim records of beneficiaries of the National Health Insurance program from 2001 to 2010 were available in the database. The 2005 LHID contains secondary data released to the public for research purposes; therefore, this study was exempted from full review by the institutional review board. Data from 2 groups of individuals were obtained: those exposed to mismatched vaccine strains during the period from approximately October 2007 to December 2007, as well as those exposed to matched strains from October 2008 to December 2008. All individuals were 65 years of age or older. Individuals diagnosed with AMI before September 2007 (for the mismatched strains) and before September 2008 (for the matched strains) were excluded, as were those diagnosed within 21 days after receiving the vaccine. For the 2007 data (mismatched strains), individuals were considered to be in the exposure cohort if their records showed that they were vaccinated against influenza during the period October 2007 to December 2007. For the 2007 analytic data, the AMI diagnosis was from January 1, 2008 to September 30, 2008. For the 2008 analytic data, AMI diagnosis was made from January 1, 2009 to September 10, 2009. Data were censored due either to death or withdrawal.

### AMI Diagnosis Co-Morbidities

The International Classification of Diseases 9th Edition Clinical Modification (ICD-9 CM) for AMI is 410. Potential co-morbidities included asthma (ICD-9 CM 493), chronic obstructive pulmonary disease (COPD; ICD-9 CM: 491, 492, 496), ischemic heart disease (ICD-9 CM: 411, 413, 414), old MI (ICD-9 CM: 412), cardiac failure (ICD-9 CM: 428), hypertension (ICD-9 CM: 401), diabetes mellitus (ICD-9 CM: 250), ischemic stroke (ICD-9 CM: 433, 434, 436), liver disease (ICD-9 CM: 571), renal disease (ICD-9 CM: 580–589), cancer (ICD-9 CM: 140–209), pneumonia (ICD-9 CM: 480–486), and influenza-like illness (ICD-9 CM: 487). The disease diagnoses were confirmed through at least 2 outpatient consultations or 1 hospitalization.

### Statistical Analysis

Two-tailed paired *t* tests were used to test the mean difference for continuous variables between the patients exposed and unexposed to the IV. Cochran Mantel–Haenszel Chi-squared tests were used to test for nominal variables. The influenza vaccination hazard risk was estimated using the Cox hazard model while adjustments were made for potential confounders. The SAS Version, 9.3 computer software (SAS Institute, Inc., Cary, NC) was used for all analyses and a *P* value < 0.05 was considered to be statistically significant.

## RESULTS

A total of 93,051 exposed and 109,007 unexposed individuals were recruited. Table 1 shows the baseline characteristics of the individuals exposed (M: 21,808; F: 22,218) and unexposed (M: 26,665; F: 29,201) to the IV in 2007. The proportional rates of the variables such as urbanization and comorbidity (asthma, COPD, ischemic heart disease, hypertension, diabetes mellitus, ischemic stroke, liver disease, influenza-like illness, and common cold) achieved statistical significance in both men and women. Table 2 shows the characteristics of the exposed (M: 25,000; F: 24,025) and unexposed (M: 28,273; F: 24,868) individuals in 2008. The proportional rates of the variables reached the level of statistical significance, similar to those in 2007. Tables 3 and 4 show the AMI risk associated with the influenza vaccinations in 2007 and 2008, estimated by gender after adjusting for age, urbanization, low income, and co-morbidities (i.e., asthma, COPD, ischemic heart disease, past MI, cardiac failure, hypertension, diabetes mellitus, ischemic stroke, liver disease, renal disease, cancer, pneumonia, influenza-like illnesses, and common cold), in the exposed and unexposed groups. The AMI hazards ratios (HRs) in the men and women exposed to mismatched strains in 2007 were 0.990 (95% confidence interval [CI], 0.745–1.316) and 1.102 (95% CI: 0.803–1.513), respectively (Table 3). As shown in Table 4, the AMI HRs were significant in the men exposed to the matched strains in 2008 (HR: 0.681; 95% CI: 0.509–0.912) and were barely significant in their female counterparts (HR: 0.737; 95% CI: 0.527–1.029).

**TABLE 1:**
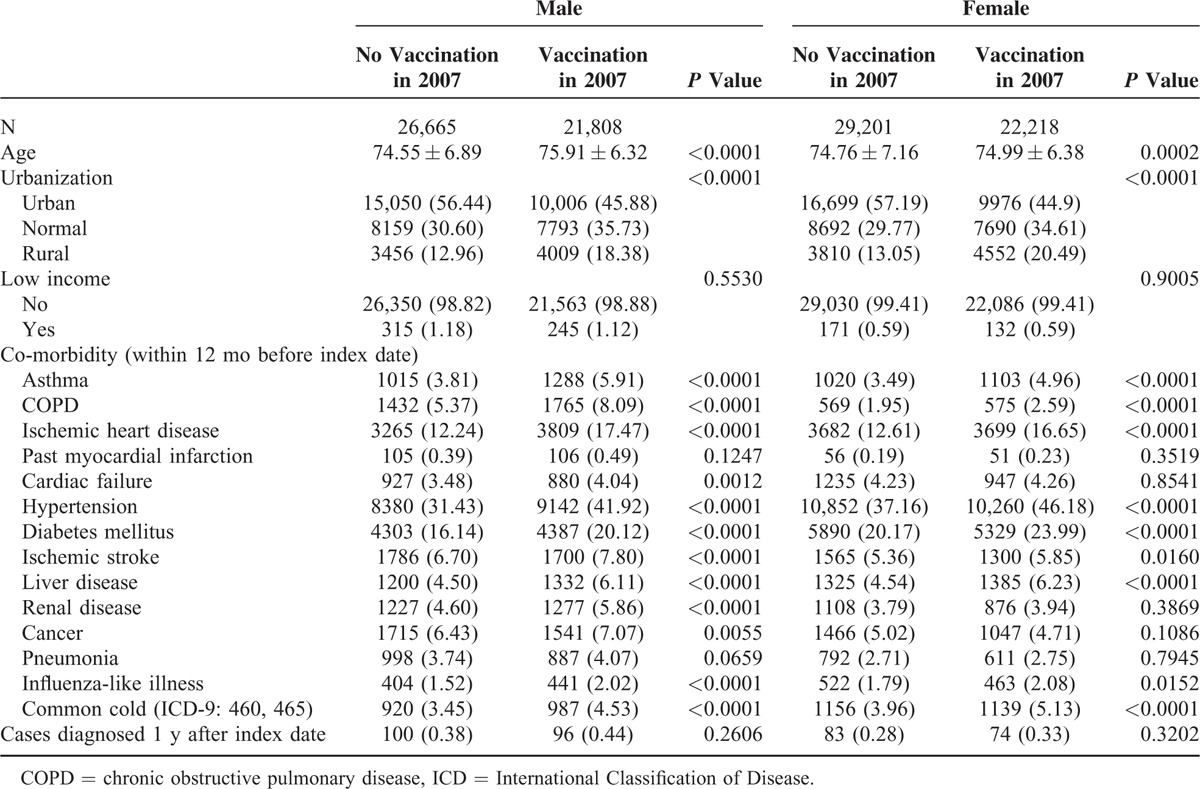
Characteristics of Individuals Exposed and Not Exposed to Influenza Vaccine in 2007

**TABLE 2:**
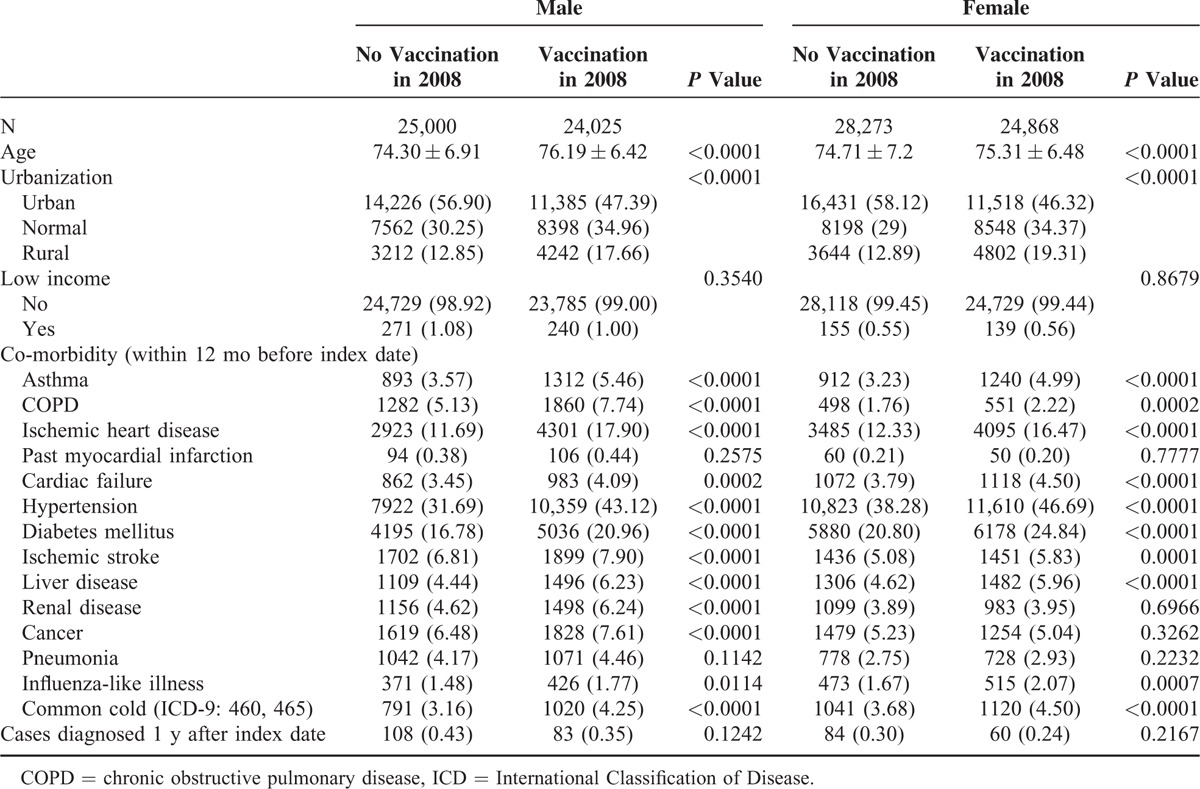
Characteristics of Individuals Exposed and Not Exposed to Influenza Vaccine in 2008

**TABLE 3:**
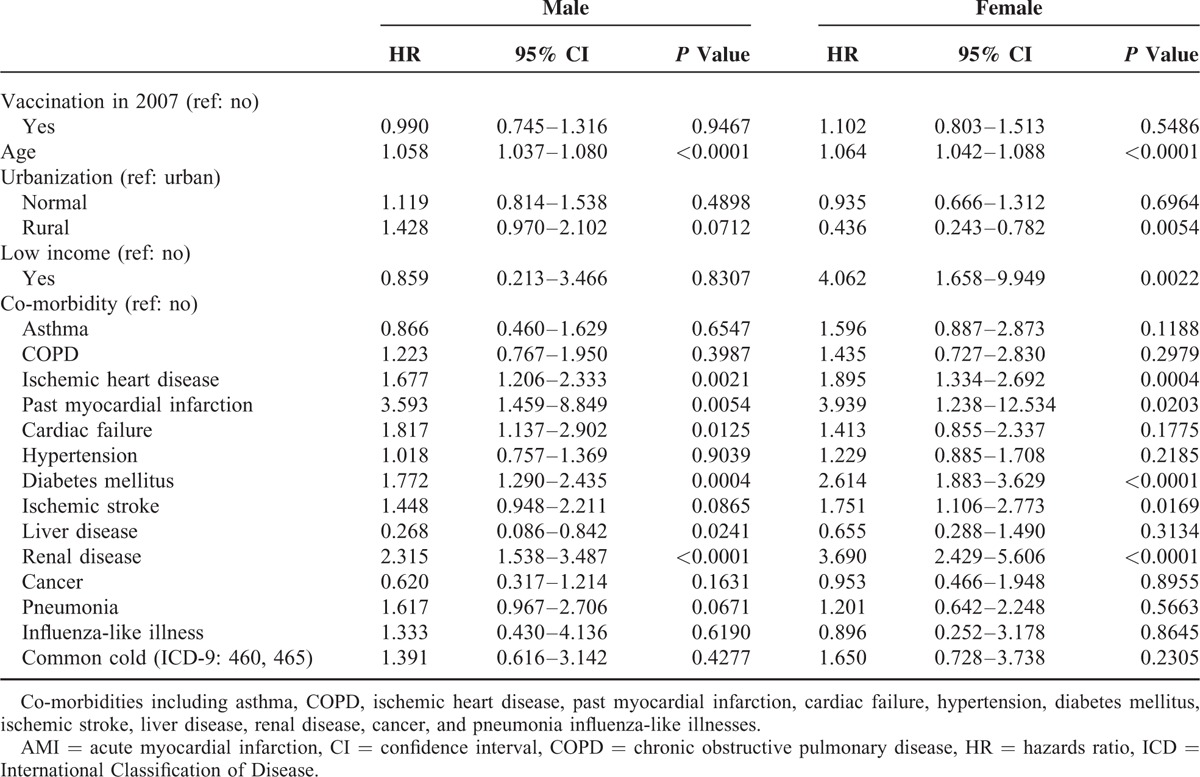
HR for AMI in the Exposed and Unexposed Individuals in 2007

**TABLE 4:**
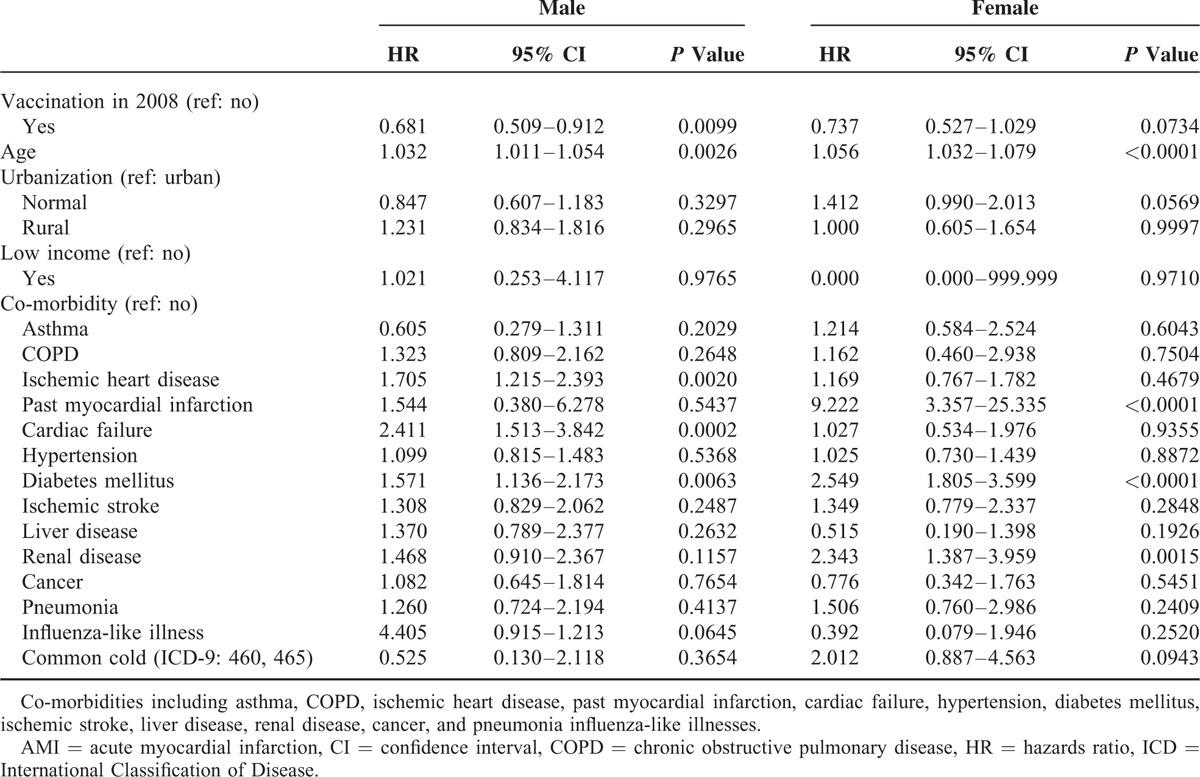
HR for AMI in the Exposed and Unexposed Individuals in 2008

## DISCUSSION

The assessment of IV effectiveness is complicated by the degree which the vaccine and virus match, which varies from year to year, with mismatches leading to contradictory results.^9^ In this study, we prospectively investigated elderly with or without influenza vaccinations, and then, compared the differences in the incidences of AMI. The results demonstrated that elderly individuals exposed to matched IV in 2008 appeared less likely to have AMI compared with those who were exposed to mismatched strains in 2007. Our results showed that elderly men who were exposed to the IV appeared to be less likely to have AMI, unlike their female counterparts in 2007 and 2008. The HRs for AMI among the individuals exposed to mismatched vaccines (administered in 2007) were 0.990 (95% CI: 0.745–1.316) for men and 1.102 (95% CI: 0.803–1.513) for women. For the matched vaccine (administered in 2008), the HRs for AMI were significant in men (HR: 0.681; 95% CI: 0.509–0.912) and barely significant in women (HR: 0.737; 95% CI: 0.527–1.029) in 2008 (match data). Few studies have investigated the association between influenza infection and AMI by gender. To the best of our knowledge, this article is the first to investigate the relationship between matched/mismatched strains of IVs and the risk of AMI by gender.

Several recent observational studies have reported reductions in AMI following influenza vaccination.^1,6,10^ Three epidemiological studies and 1 small clinical trial have suggested that influenza vaccination is associated with a 50% reduction in the incidence of sudden cardiac death, AMI, and ischemic stroke.^11^ However, previous randomized clinical trials have demonstrated inconclusive effects for cardiovascular death.^12,13^ Although the “health vaccine effect” could not be ruled out, the main findings in Gwini's work align with our study.^10^ However, the “healthy vaccine effect” was minimized in the study design of this article. The vaccine only provides only between 70% and 80% protection against influenza virus strains that closely match those in the vaccine, but the level of effectiveness of the vaccine varies, and it is slightly lower (43–68%) in the elderly.^14,15^ None of the previous papers have investigated the relationship between matched/mismatched strains of IVs and the risk of AMI.

Previous studies have also suggested that the use of IV was associated with a lower risk of major adverse cardiovascular events,^6,16^ and Meyers^11^ has also shown that AMIs could be prevented through influenza vaccinations. Phrommintikul et al^5^ conducted a study to evaluate the effects of the IV on cardiovascular outcomes in acute coronary syndrome (ACS) patients; the authors reported that the IV reduced major cardiovascular events in patients with ACS. Gwini et al^10^ also investigated the association between AMI and influenza vaccination using the self-controlled case-series method, and found that reductions in AMI incidence were more pronounced for early seasonal vaccinations before mid-November. These previous studies have used either ACS patients^5^ or a self-controlled case-series study design^10^ that was consistent with ours. However, negative studies^6,10–12^ and equivocal conclusions from a recent systematic review^10,11^ indicate that there is insufficient and conflicting evidence, particularly in representative, low risk populations. A review paper of Keller et al^2^ has also concluded that insufficient data exist to evaluate the effect of vaccination on coronary heart disease. Therefore, the protective effects of the IV on cardiovascular events are still inconclusive because several confounding variables could be adjusted. These potential confounders might include vaccine type (match or mismatch), gender, underlying diseases, and different outcomes measured. Nichol et al^17^ conducted a large-scale study that included, cohorts of community-dwelling members of 3 large managed-care organizations who were at least 65 years old; these residents were studied during the 1998 to 1999 and 1999 to 2000 influenza seasons. The authors found that in the elderly, the influenza is associated with reductions in the risk of hospitalization for heart disease, cerebrovascular disease, and pneumonia or influenza as well as the risk of death from all causes during influenza seasons. This result aligns with our results. However, in Nichol's work,^17^ the outcome is not specific to AMI outcome, nor is it stratified by gender; the misclassification of vaccination status and vaccine type (match or mismatch) is other concerns. In this study, we investigate the relationship between matched/mismatched strains of IVs and the risk of AMI by gender using large-scale population-based data to conduct a retrospective cohort study.

The current IV used worldwide contains 3 different virus strains including 2 type A virus (H1N1 and H3N2) and 1 type B virus. It has been widely accepted that IVs are most effective when there is a good match between circulating viruses and vaccine strains; protection may also be substantial, although sometimes lower, during the years with a poor match among healthy adults and elderly individuals who are at high risk or institutionalized. In this study, we selected 2 different influenza seasons for which it was previously suggested that the circulating virus did or did not match with the vaccine strains for all 3 types of influenza viruses in 2008 and 2007, respectively. Furthermore, adjustments were made for age, urbanization, low income, potential co-morbidities (asthma, COPD, ischemic heart disease, past MI, cardiac failure, hypertension, diabetes mellitus, ischemic stroke, liver disease, renal disease, cancer, and pneumonia), and influenza-like illness. Our results confirmed that the protective effect of IV was more profound among the elderly who received a matched IV in 2008 compared with those in 2007 who received mismatched strains in 2007. AMI remains leading causes of death worldwide. The results of this study could be used to support public health primary prevention programs for AMI particularly among the elderly.

In a previous case–control study to test the association between IV infection and acute cardiovascular conditions, Guan et al^18^ investigated whether the presence of IgG antibody to IV-A and IV-B was associated with AMI. This study supported the hypothesis that previous IV infection affected the development of atherosclerosis and triggered the occurrence of AMI.^18^ The mechanism through which IV infection leads to AMI is unclear. To explore the mechanism of atherosclerosis, Guan et al^19^ conducted a case–control study to examine inflammatory cytokines to assess the association between previous IV infection and AMI, and they found that inflammatory cytokines may participate in the development of atherosclerosis and trigger the occurrence of AMI. A self-controlled case series analysis indicated that influenza infection can act as a trigger for AMI.^20^ In this study, we did not test the association between the influenza virus (IV) and AMI. However, the current results demonstrated that elderly individuals exposed to matched IV in 2008 appeared to be less likely to have AMI, unlike those who were exposed to mismatched strains in 2007. This study is the first to imply that elderly individuals who are exposed to matched IVs with the strength to protect against IV have a lower risk of AMI. This finding highlights the benefits and importance of matched IVs and supports efforts to increase the rates of matched vaccination among the elderly.

This study has a limitation. We did not investigate the circulating influence of virus titers in the blood of the study participants to correlate the presence of influenza virus and the triggering of AMI episodes. The effect between vaccine and AMI risk could be well understood through our study. AMI risk could be reduced, particularly in men, if the IV strain matches the circulating strains in elderly men over the age of 65. The mechanisms explaining such gender differences deserve further study. More studies are needed to evaluate the relationship between AMI risk and matched IV in men 65 years of age and younger.

## CONCLUSIONS

AMI risk could be reduced if matched IVs are used, particularly in men 65 years and older.
